# Nasal histological findings in asymptomatic control dogs and in dogs with chronic inflammatory rhinitis

**DOI:** 10.1177/03009858251349138

**Published:** 2025-06-24

**Authors:** Henriikka Neittaanmäki, Hanna-Maaria Javela, Essi Kuningas, Katja Koskinen, Anni Tilamaa, Minna Rajamäki, Sanna Viitanen, Niina Airas

**Affiliations:** 1 University of Helsinki, Helsinki, Finland

**Keywords:** canine, chronic inflammatory rhinitis, histopathology, idiopathic inflammatory rhinitis, lymphoplasmacytic rhinitis, nasal disease

## Abstract

Chronic inflammatory rhinitis (CIR) is among the most common causes of chronic nasal signs in dogs. Despite research efforts, the etiology of CIR remains mostly undiscovered. The aim of our study was to describe the histological findings in nasal biopsies of control dogs without signs of nasal disease compared to dogs with CIR. The study groups were control dogs euthanized for reasons unrelated to this study (*n* = 20) and previously collected, archived nasal biopsies from dogs diagnosed with CIR (*n* = 20). A CIR diagnosis was based on clinical presentation, computed tomography, rhinoscopy, and histopathological findings indicative of CIR. Inflammatory cell counts and changes in the mucosal epithelium and associated lamina propria were evaluated from nasal biopsy specimens. The numbers of lymphocytes and plasma cells (*P* < .0001), neutrophils (*P* < .0001), and eosinophils (*P* = .0016) in the lamina propria, and mucosal intraepithelial leukocytes (*P* < .0001) were significantly higher in dogs with CIR compared to control dogs. A small population of leukocytes was also observed in control dogs, likely representing a physiological immune cell population. The type of inflammation in CIR is not purely lymphoplasmacytic, as both neutrophils and eosinophils were also detected in CIR dogs. The mucosal epithelium was thicker (*P* = .006), and visible goblet cells (*P* < .001) were decreased, in dogs with CIR, with a multifocal loss of cilia in some dogs, which may represent a form of respiratory epithelial metaplasia. Epithelial alterations likely play a role in the pathophysiology of CIR and contribute to the clinical signs.

Chronic inflammatory rhinitis (CIR, also called canine idiopathic lymphoplasmacytic rhinitis) is among the most common causes of chronic nasal signs in dogs and is diagnosed in 24 to 39% of cases.^17,24,32^ The most common clinical signs comprise chronic nasal discharge, which is most often bilateral and mucopurulent, and sneezing.^16,37^ Varying degrees of nasal turbinate destruction have been described in affected dogs.^16,32,37^ A CIR diagnosis is obtained through extensive clinical examinations to exclude other causes for chronic nasal signs, such as nasal neoplasia, infectious etiologies, and dental disease, and by demonstrating predominantly lymphoplasmacytic inflammation in nasal biopsies.^29,37^ CIR is a frustrating, mostly incurable disease lacking consistently effective treatments, thus affecting the quality of life of both dog and owner.

Despite research efforts, the etiology of CIR remains mostly undiscovered. Dysregulation of the local immune system, initiated by an unknown insult and resulting in mucosal damage, further inflammation, and secondary infections, is suspected to underlie the disease,^7^ but the evidence supporting these suspicions is lacking. An increased expression of toll-like receptors 1, 2, and 6–8, along with distinct cytokine and chemokine proﬁles, has been demonstrated in dogs with CIR when compared to healthy control dogs.^18,23^ The immune response in CIR is characterized by an increase in *interleukin-5* mRNA expression that is distinct from the immune response demonstrated in sinonasal aspergillosis.^23,35^ The role of specific pathogens has been explored, and *Bartonella sp., Mycoplasma sp.*, and *Chlamydophila sp.* were not detected in the nasal biopsies of affected dogs.^12,38^ High levels of fungal DNA have been demonstrated in the nasal biopsies of CIR dogs, which has been suggested as a possible underlying or perpetuating factor; however, the accumulation or entrapment of fungi secondary to inflammation may also explain the fungal DNA detected.^38^ CIR may be multifactorial, and both odontogenic infections and gastrointestinal disease or gastro-esophageal reflux have been suggested to contribute to the disease.^8,10,29^ A better understanding of the etiology of CIR could aid in the identification of possible risk factors and the planning of therapeutic interventions.

Histopathological findings in CIR include infiltration of the fibrotic stroma by lymphocytes and plasma cells and, to a lesser extent, neutrophils, and variable hyperplasia, erosion, or squamous metaplasia of the surface epithelium.^7^ The inflammatory findings in CIR are not pathognomonic, as similar histopathological findings have been described in both CIR and in sinonasal aspergillosis.^9^ In addition, inflammatory changes do not always correlate with clinical signs, as mild histological changes can remain after treatment even with complete clinical resolution of CIR signs.^13^ To our knowledge, no studies report the histological findings in the nasal mucosa of healthy dogs. Since the nasal cavity is constantly in contact with inhaled microbes and particles, it is of interest to describe the nasal histology in asymptomatic dogs and to compare the findings to those with CIR to better understand the clinical relevance of pathophysiological changes occurring in CIR.

The aim of our study was to describe the histological findings in the nasal biopsies of dogs without signs of nasal disease compared to dogs with CIR.

## Materials and Methods

### Case Collection

Privately owned dogs thoroughly examined for chronic nasal signs at the Veterinary Teaching Hospital of the University of Helsinki between 2013 and 2023 were retrospectively reviewed, and 20 dogs diagnosed with CIR were included in the study. Archived paraffin-embedded biopsy samples from dogs diagnosed with CIR were used in the study. In addition, nasal biopsies were prospectively obtained from privately owned control dogs without nasal signs that were euthanized for reasons unrelated to this study and donated for research purposes between March 2023 and November 2023.

All dogs with CIR had undergone thorough clinical examinations including computed tomography (CT) with and without iodine contrast, along with antegrade and retrograde rhinoscopy with biopsy acquisition under general anesthesia. Biopsies were obtained either blindly or with endoscopic guidance from both nasal cavities using single-use biopsy forceps. A CIR diagnosis was made when chronic nasal signs (nasal discharge +/- sneezing or reverse sneezing) were present for at least 2 months, CT and rhinoscopy findings were indicative of CIR, a predominantly lymphoplasmacytic inflammation was observed in nasal biopsies, and other causes for nasal signs were excluded.^37^ Dogs were excluded if the inflammation was predominantly eosinophilic, the nasal discharge was solely epistaxis, or if CT findings indicated mass lesions or inflammation in the maxillary teeth roots, as well as in the case of masses, foreign bodies, or fungal plaques detected with CT or rhinoscopy. In addition, dogs with inadequate nasal biopsies were excluded. Biopsy specimens were considered inadequate if they were very small or if the diagnostic quality was poor due to artifacts being present. At least 2 high-powered fields (2 times 0.237 mm^2^) with intact mucosal epithelium and lamina propria without artifacts were needed for inclusion. After the first review, 33 dogs with CIR were considered, and in the end, 20 dogs best fitting the criteria were included in the study.

All owners of asymptomatic control dogs were questioned concerning possible nasal signs and concurrent medications at the time of euthanasia, and the responses were recorded. Dogs were included in the study if all nasal signs were absent (nasal discharge, sneezing, and reverse sneezing) and in the absence of medications possibly affecting the inflammatory response (antimicrobials, nonsteroidal anti-inflammatory drugs, and corticosteroids). Nasal biopsies were obtained blindly immediately after euthanasia from the middle compartment of both nasal cavities with single-use biopsy forceps. After biopsy acquisition, a complete postmortem examination was performed including evaluation of the entire nasal cavities to rule out subclinical nasal disease.

### Computed Tomography Grading

CT scans were evaluated and graded by a single radiologist (AT). Soft tissue density indicative of secretion in the nasal cavity and nasal turbinate destruction were graded as none, mild, moderate, or severe (0–3) in rostral, middle, and caudal parts of both nasal cavities. The total score (0–18) of each variable was used for statistical analysis.

### Histology

Nasal biopsy specimens were fixed in 10% neutral-buffered formalin, routinely processed, and stained with hematoxylin and eosin, Masson trichrome, periodic acid-Schiff, and Gram. Each biopsy sample was evaluated by NA (a board-certified pathologist) and HN. At the time of histological evaluation, the pathologists were blinded to the patient data.

The numbers of inflammatory cells in the nasal mucosa were calculated, including the mucosal epithelium and associated lamina propria. The numbers of cells in the lamina propria were calculated as lymphocytes and plasma cells, neutrophils, and eosinophils for 3 high-power (40×) fields (0.237 mm^2^) per each side of the nasal cavity. The total number of mucosal intraepithelial leukocytes was calculated from the same fields. The 3 fields were selected randomly from the areas of best diagnostic quality. Evaluation of different intraepithelial inflammatory cell types was performed by counting 10 randomly selected intraepithelial inflammatory cells per dog. Epithelial thickness was evaluated from the same 3 high-power fields as the inflammatory cell count and calculated as epithelial cell layers. The lamina propria including the mucosal glands was further evaluated. The specimens were graded for the presence of edema in the lamina propria, lymphoid hyperplasia, and fibrosis. The mucosal epithelium was evaluated for epithelial damage and number of goblet cells. The presence of fungi, bacteria, intravascular leukocytes, and vasculitis was recorded as present or absent. The presence of cartilaginous and/or osseous tissue was recorded, and it was evaluated for inflammatory and structural changes. The presence of mucus was recorded. Due to the small size and/or damage to the biopsy specimens, evaluating 3 representative high-power fields was impossible in 3 CIR dogs and 1 control dog, and 2 fields were assessed instead. In these dogs, the estimated cell count of 3 high-power fields was calculated based on the available fields (the average cell count in the available fields was multiplied by 3).

The basis of the grading is presented in Table 1, and a detailed description of grading criteria is described in Supplemental Table S1. For each variable, the mean value from the 2 nasal cavities was used in the statistical analysis.

**Table 1.** Histopathological grading of the nasal biopsy specimens.

Factor	Grading	Basis of grading
Lamina propria leukocytes * Lymphocytes—plasma cells* * Neutrophils* * Eosinophils*	Continuous	Absolute number / 3 HPF (0.237 mm^2^)
Mucosal intraepithelial leukocytes	Continuous	Absolute number / 3 HPF and differentiation of 10 cells per specimen
Epithelial thickness	Continuous	Epithelial cell layers, mean from 3 HPF
Edema in the lamina propria	0 absent 1 mild (< 25%) 2 moderate (25-50%) 3 severe (> 50%)	Percentage of the specimen area having edema
Lymphoid hyperplasia	0 absent 1 mild (< 25%) 2 moderate (25-50%) 3 severe (> 50%)	Percentage of the specimen area having lymphoid hyperplasia
Fibrosis	0 absent 1 mild 2 moderate 3 severe	Presence of fibrosis on the Masson trichrome-stained section based on the width of the fibrotic stroma and the presence of mucosal gland atrophy
Epithelial damage	0 absent 1 erosion 2 ulceration	(1) Mild epithelial damage, (2) damage extending through the basal cell layer and causing local inflammation
Goblet cells	0 absent 1 <5 per HPF 2 5-10 per HPF 3 >10 per HPF	Frequency of visible goblet cells per HPF across the specimen on the PAS stain
Fungi	0 absent 1 present	Presence of fungi on the PAS stain
Bacteria	0 absent 1 present	Presence of bacteria on the Gram stain
Intravascular leukocytes	0 absent 1 present	Filling of capillaries with leukocytes
Vasculitis	0 absent 1 present	Inflammatory cells in the capillary walls with endothelial damage and surrounding edema
Cartilaginous/osseous tissue	0 absent 1 present, normal 2 present, abnormal	Presence of cartilaginous/osseous tissue and evaluation of inflammatory/structural changes
Mucus	0 absent 1 present	Presence of mucus and evaluation of possible inflammatory infiltrate

Abbreviations: HPF, high-power field; PAS, periodic acid-Schiff.

### Immunohistochemistry

Immunohistochemistry was performed to further characterize the nasal lymphocyte population. Nasal biopsy specimens were first incubated for 20 minutes at 99°C in 10 mM citrate buffer (pH 6) for heat-induced antigen retrieval. Endogenous peroxidase was quenched by immersion in 3% hydrogen peroxide for 10 minutes, and nonspecific antibody binding was blocked using 10% bovine serum albumin (BSA, Merck, Darmstadt, Germany) in phosphate-buffered saline. The primary rabbit polyclonal CD3 (1:200, A0452, Agilent/Dako) and mouse monoclonal CD79a (1:400, MCA2538H, AbDSerotec) antibodies were incubated at room temperature for 60 minutes. The secondary antibody, polymer-linked to HRP (BrightVision + Poly-HRP kit; ImmunoLogic, Duiven, The Netherlands), was incubated in a humid chamber for 30 minutes at room temperature, and the immunoreaction was visualized with the Bright DAB Substrate kit (ImmunoLogic, Duiven, Netherlands). Canine tissues from routine diagnostic cases were used as positive controls, and the absence of nonspecific binding was shown by omitting the primary antibody.

Both immunohistochemical assays were evaluated in the same areas where lymphocytes were present. T and B lymphocytes were calculated for 2 high-power fields (0.237 mm^2^) per each side of the nose.

### Statistical Analysis

Data normality was explored using the Shapiro-Wilk test and visual inspection of normality plots. Differences between CIR dogs and controls were assessed with the Mann-Whitney *U*-test. Correlations between clinical and histopathological variables were assessed for CIR dogs using Spearman correlation coefficients. Moderate correlation was detected when *r* = 0.38–0.67 and strong correlation when *r* = 0.68–0.87. Statistical analyses were performed using commercial statistical software (GraphPad Prism 10.1.2, Dotmatics, Boston, Massachusetts). *P*-values < .05 were considered statistically significant.

### Ethical Approval and Owner Consent

The use of surplus paraffin-embedded biopsy samples from dogs with CIR was not subjected to ethical review. At the Faculty of Veterinary Medicine, University of Helsinki, the Pathology and Parasitology Unit holds all further rights to histopathological specimens submitted for analysis. Clients are informed of this faculty policy in writing. The use of client-owned cadavers donated for research purposes has been approved by the Viikki Campus Research Ethics Committee of the University of Helsinki, Finland (Statement 17/2021).

The histological grading of each dog is available in Supplemental Table S2, and the full data analyzed in this study are available from the corresponding author upon reasonable request.

## Results

### Study Population

Study groups comprised previously collected archived nasal biopsies from 20 dogs diagnosed with CIR and 20 prospectively collected control dogs without nasal signs. Dogs with CIR were younger (*P* = .005, median age = 7.3 years, interquartile range [IQR] 6–9.75, range = 2.5–12 years at the time of biopsy) than control dogs (11.3 years, IQR = 8.1–12.6, range = 4.5–16.8 years). Dogs with CIR were also smaller (*P* = .03, median weight = 10.1 kg, IQR = 7.3–17.9, range = 6–47.6 kg) than the controls (median weight = 21.2 kg, IQR = 12.4–31.3, range = 5–54 kg).

Dogs with CIR comprised 7 dachshunds, 4 Parson Russell terriers, 2 whippets, 2 mixed-breed dogs, and 1 of each: Miniature pinscher, American Akita, briard, border collie, and Mittelspitz. Control dogs comprised 6 mixed-breed dogs, 2 dachshunds, 2 border collies, and 1 of each: Karelian bear dog, grand basset Griffon Vendéen, Belgian shepherd, Borzoi, giant schnauzer, Keeshond, rottweiler, Icelandic sheepdog, golden retriever, and Cavalier King Charles Spaniel.

The cause for euthanasia in all control dogs was unrelated to nasal diseases and most commonly was a deterioration of the general condition due to acute or chronic illness. None of the control dogs exhibited nasal signs or received medications capable of altering immune responses. A full description of the demographics and clinical signs of CIR dogs is provided in Supplemental Table S3, and the demographics, cause for euthanasia, and main postmortem diagnosis of control dogs are provided in Supplemental Table S4.

### Clinical Findings

Clinical signs in dogs with CIR included nasal discharge in all the dogs (serous 5/20, mucopurulent 19/20; bilateral 18/20, unilateral 2/20), sneezing in 18/20 dogs, and reverse sneezing in 12/20 dogs. The median duration of clinical signs was 8 months (IQR = 5.5–12, range = 2–36 months). The severity of clinical signs was not systematically recorded in patient records; therefore, only the duration of clinical signs was included in the statistical analysis.

CT imaging revealed none (3/20), mild (6/20), or moderate (11/20) soft tissue densities indicative of secretion in the nasal cavity, and none (14/20), mild (4/20), or moderate (2/20) turbinate destruction. The CT findings did not correlate significantly with the severity of the inflammatory changes in the nasal biopsies or with clinical sign duration. Rhinoscopy findings included normal conchae structure (20/20), nasal secretions (18/20), hyperemia (7/20), and mild nasal turbinate destructions (3/20). The findings were bilateral in 18/20 and unilateral in 2/20 dogs.

The postmortem examination findings in control dogs included a variety of neoplasia, osteoarthrosis, and many nonspecific findings (Supplemental Table S4). None of the control dogs had macroscopic lesions in their nasal cavities.

### Histopathological Findings

**Figure 1.**
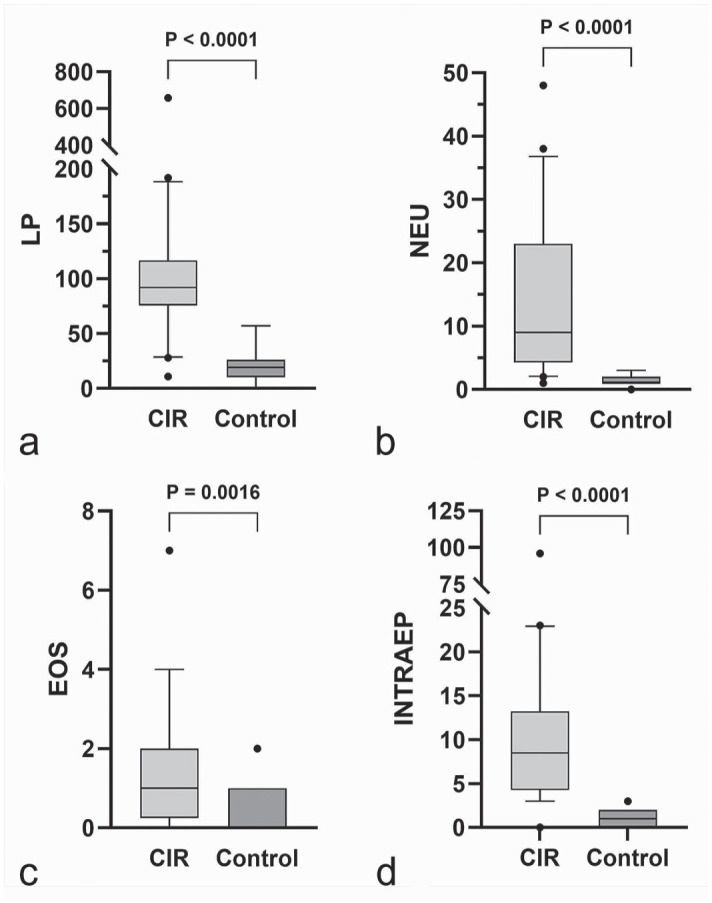
Box and whisker plots presenting medians and interquartile ranges of (a) lymphocyte and plasma cell (LP), (b) neutrophil (NEU), and (c) eosinophil (EOS) counts in the lamina propria, and (d) intraepithelial leukocyte cell (INTRAEP) counts of 3 random high-power fields in nasal histological specimens from dogs with chronic inflammatory rhinitis (CIR, *n* = 20) and from control dogs (*n* = 20). Dots are values outside the 10–90 percentiles. Cell counts are significantly higher in CIR compared to controls.

**Figure 2.**
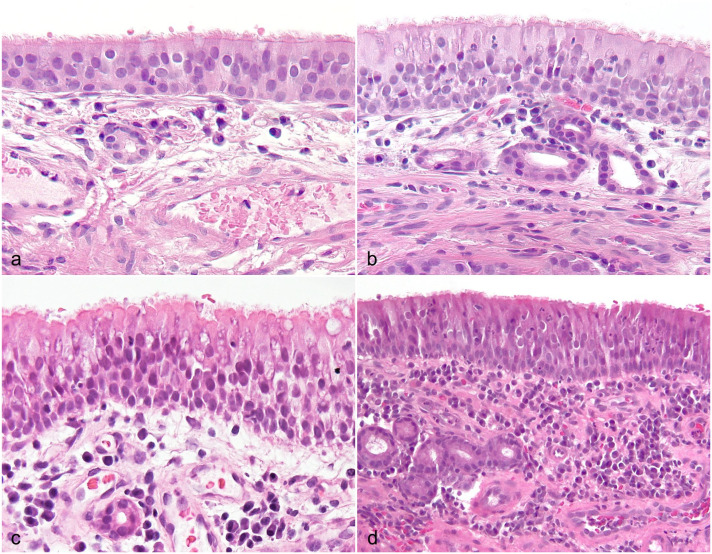
Nasal histological specimens from (a) a control dog without nasal signs and from (b–d) dogs with chronic inflammatory rhinitis. There are variable numbers of leukocytes in the lamina propria (similar in a and b, increasing in c and d) and in the mucosal epithelium, edema in the lamina propria (c), and intravascular neutrophils (d). Hematoxylin and eosin.

The numbers of lymphocytes and plasma cells (*P* < .0001), neutrophils (*P* < .0001), and eosinophils (*P* = .0016) in the lamina propria were significantly higher in dogs with CIR compared to control dogs (Fig. 1a–c). The main inflammatory cell population in CIR was lymphocytes and plasma cells in all samples. Still, great variation was observed in the severity of inflammatory infiltration in both CIR and control dogs (Fig. 2). Lymphocytes and plasma cells were found in the lamina propria of all the control dogs, and they were present bilaterally in all dogs, although the number of cells differed (>50%) between the left and the right side in 6/20 of the CIR dogs and in 7/20 of the control dogs. Lymphocytes and plasma cells were diffusely distributed but were most frequent in the superficial lamina propria right under the epithelium, with multifocal variation especially in CIR dogs. Neutrophils were present in all CIR dogs and in 19/20 control dogs, 11 of which had only 1 neutrophil present in 3 high-power fields. Eosinophils were present in 15/20 CIR dogs and 6/20 control dogs. Moderate positive correlations were found between lymphocyte and plasma cell counts vs neutrophil counts (*r* = 0.621, *P* = .003) and between neutrophil and eosinophil counts (*r* = 0.547, *P* = .013).

The number of mucosal intraepithelial inflammatory cells was higher in dogs with CIR (*P* < .0001, Fig. 1d), and it was positively correlated with inflammation in the lamina propria (lymphocytes and plasma cells *r* = 0.676, *P* = .001; neutrophils *r* = 0.747, *P* < .0001; eosinophils *r* = 0.496, *P* = .0026) and epithelial thickness (*r* = 0.541, *P* = .0014). In dogs with CIR, 91/200 (45%) of the evaluated intraepithelial inflammatory cells were lymphocytes and 109/200 (55%) were neutrophils, whereas in control dogs, 184/200 (92%) were lymphocytes and 16/200 (8%) were neutrophils. Intraepithelial neutrophils were positively correlated with neutrophils in the lamina propria (*r* = 0.489, *P* = .029) in dogs with CIR. Intraepithelial eosinophils were not found.

Intravascular leukocytes were all found to be neutrophils (Fig. 2d), so the term intravascular neutrophils is used from now on. Intravascular neutrophils (*P* < .001) and vasculitis (*P* = .009) were also more common in CIR; they were present in 15/20 and 6/20 CIR dogs and in 1/20 and 0/20 of the control dogs, respectively. Moderate correlations were found between intravascular neutrophils and all the inflammatory cell counts in the lamina propria including lymphocyte and plasma cells (*r* = 0.471, *P* = .036), neutrophils (*r* = 0.552, *P* = .012), and eosinophils (*r* = 0.652, *P* = .002).

**Figure 3.**
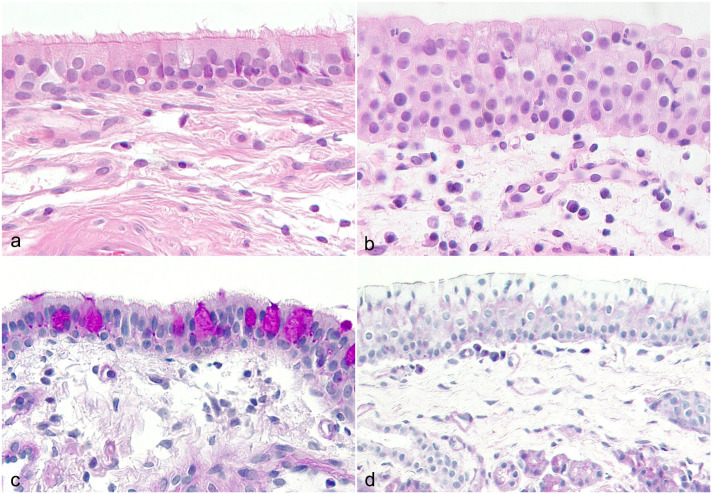
Nasal histological specimens from control dogs without nasal signs and from dogs with chronic inflammatory rhinitis (CIR) presenting the mucosal epithelial thickness and structure. (a) Normal mucosal epithelial structure in a control dog without nasal signs. Hematoxylin and eosin (HE). (b) Thickened mucosal epithelium with possible metaplasia and loss of cilia, edema in the lamina propria, and a reactive vessel in a dog with CIR. HE. (c) Goblet cells are present in the mucosal epithelium in a control dog. Periodic acid-Schiff (PAS). (d) No visible goblet cells are present in the dog with CIR. PAS.

**Figure 4.**
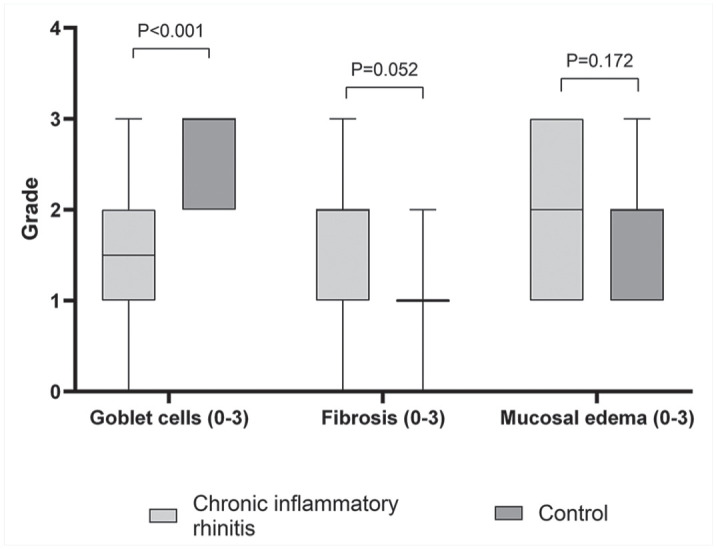
Box and whisker plots presenting medians and interquartile ranges of goblet cell, fibrosis, and mucosal edema grades (graded from 0 to 3) in nasal histological specimens from dogs with chronic inflammatory rhinitis (*n* = 20) and from control dogs (*n* = 20).

The nasal mucosal epithelium was thicker in CIR dogs than in control dogs (*P* = .006, Fig. 3a, b). The median number of epithelial cell layers was 4 (IQR = 3.5–5, range = 3–8) in CIR and 3 (IQR = 3–4, range = 2–6) in control dogs. Dogs with CIR had fewer visible goblet cells than control dogs did (*P* < .001, Figs. 3c, d and 4). Fibrosis was more common in CIR dogs (*P* = .052), but this did not reach statistical significance (Fig. 4). Negative correlations were found between goblet cells and epithelial thickness (*r* = −0.493, *P* = .027) and between goblet cells and fibrosis (*r* = −0.647, *P* = .002), with fewer goblet cells being present when the epithelium was thicker and when more fibrosis was present. There was a multifocal lack of cilia in 8/20 of the CIR dogs, while the cilia were normal in 12/20 CIR dogs and in all control dogs.

No significant differences were found in edema in the lamina propria (*P* = .172), epithelial damage (*P* = .403), lymphoid hyperplasia (*P* = .435), or the presence of bacteria between the groups (*P* = .685). Lymphoid hyperplasia was present in 5/20 CIR dogs and in 3/20 control dogs and was mild in all dogs. Correlation was not found between lymphoid hyperplasia and the age of the dog (*r* = −0.261, *P* = .267). Bacteria were only found on the epithelial surface or detached parts of the epithelium in 4/20 CIR dogs and 3/20 control dogs. Moderate negative correlations were found between bacteria and neutrophils (*r* = −0.500, *P* = .025), intraepithelial leukocytes (*r* = −0.467, *P* = .038), and the epithelial thickness (*r* = −0.529, *P* = .016). A positive correlation was found between bacteria and epithelial damage (*r* = 0.531, *P* = .016). No fungal structures were found in the hematoxylin and eosin or the periodic acid-Schiff stained sections of any of the specimens, as expected in CIR dogs based on diagnostic criteria.

Cartilaginous/osseous tissue was present in the biopsies in 18/20 CIR dogs and in 20/20 control dogs. Inflammation did not extend into cartilaginous/osseous tissue in any of the dogs. No structural changes were found in the cartilaginous/osseous tissue. However, they were often present as very small fragments, which hindered us from evaluating their structure properly. Mucus was present in 8/20 CIR dogs and in 4/20 control dogs, and 2 dogs in each group had inflammatory infiltrates present in the mucus. The presence of mucus in CIR dogs might be affected by the saline lavage often used during rhinoscopy.

### Immunohistochemical Findings

Both groups had a heterogeneous population of lymphocytes. In CIR dogs, 52% were CD3+ T lymphocytes and 48% were CD79a+ B lymphocytes. In control dogs, 56% were CD3+ T lymphocytes and 44% were CD79a+ B lymphocytes. T lymphocytes were located in both the mucosal epithelium and lamina propria, whereas B lymphocytes were located only in the lamina propria.

## Discussion

CIR is a frustrating clinical disease with poor response to medical treatment, and a better understanding of the etiology and pathophysiology is highly warranted. The present study provides a detailed description of the histological findings in the nasal biopsies of control dogs without nasal signs and describes the changes detected in dogs with CIR, with similar criteria allowing direct comparison. These findings enable a more precise assessment of what may be considered normal in the nasal mucosa, add to the knowledge of histopathological changes developing in CIR, and eventually offer a basis for future etiological studies regarding CIR.

CIR has most commonly been reported in large, mesocephalic breeds.^29,36,37^ Contrary to this, most dogs diagnosed with CIR in our study were of small breeds. This could represent a change in the disease characteristics over time, as the previous studies were published almost 20 years ago, or geographic areas could show differences in the disease phenotype. Moreover, all these studies were relatively small, so the differences detected may also be coincidental. Dachshunds were overrepresented (7/20 CIR dogs) in our study, which could support the previously suggested breed predisposition.^15,16,29^ The overrepresentation of CIR in dachshunds has been suspected to be caused by periodontal disease and dolichocephalic conformation.^15,29^ However, all CIR dogs with tooth root alterations were excluded in our study, indicating that dental disease is not solely responsible for the breed predisposition to CIR development. Dolichocephalic conformation may be linked to CIR, as 9/20 of the affected dogs in our study belonged to dolichocephalic breeds (dachshund, whippet). Control dogs were significantly older than dogs with CIR, which reflects the typical age of euthanasia in client-owned pet dogs, and this could lead to overestimation of age-related nasal mucosal changes. However, the authors consider that this was unlikely to affect the results of this study, as our control population showed minimal nasal mucosal changes.

We found leukocytes present in the nasal lamina propria in both groups. The small population of leukocytes, mainly lymphocytes and plasma cells, seen in the lamina propria of control dogs without nasal signs likely represents a physiological immune cell population. This was expected, as the nasal mucosa is constantly in direct contact with inhaled microbes and particles, and the finding is in line with previous reports describing small numbers of leukocytes also in healthy human nasal mucosa.^30^ The number of lymphocytes and plasma cells was markedly increased in CIR. This finding is in line with other studies reporting lymphoplasmacytic inflammation in CIR;^9,37^ however, an overlap remained in the numbers of lymphocytes and plasma cells between the 2 groups, and a great variation in the severity of lymphoplasmacytic inflammation was detected in CIR. This may be caused by multifocal lesions with biopsy sampling performed in a less affected area or fluctuation in the natural course of the disease. In addition, in some dogs, clinical signs may be caused by factors other than inflammatory changes, as epithelial alterations may contribute to the clinical signs.

While lymphocytes and plasma cells were the largest inflammatory cell population in dogs with rhinitis, the number of neutrophils was also increased. This finding has been reported previously in dogs with this disease and in human chronic rhinosinusitis.^21,23,37^ No significant difference was found in the presence of bacteria between the groups, so we were unable to connect neutrophilic infiltration with bacterial infection. No correlation was found between neutrophil infiltration and epithelial damage, and the number of neutrophils was negatively correlated with the presence of bacteria. These were unexpected findings, as neutrophil infiltration was expected to accompany epithelial damage.^1^ In addition, secondary bacterial infection has been considered a clinically important feature of CIR, and a response to antimicrobial treatment is commonly described.^37^ These findings may be explained by relatively mild changes in affected dogs and that our study may have been underpowered to detect these subtle differences. Furthermore, we only detected visible bacteria on the epithelial surface, and it is established that mucosal bacteria may be difficult to detect with conventional methods.^6,14^ Therefore, the role of possible mucosal bacterial infections cannot be ruled out in our study and should be further evaluated with molecular methods with a better sensitivity. On the contrary, the commonly reported response to certain antimicrobials, such as doxycycline, in dogs with CIR could be due to other nonantimicrobial actions affecting inflammation and proteolysis.^26^ The complexity of the interplay between bacteria and mucosal inflammatory responses has been investigated in humans with chronic rhinosinusitis, and it appears that mucosal bacteria may both evade and modulate the local immune response.^14,38,41^ In dogs, it has been shown that the nasal microbiome is altered in CIR,^33,34^ but the relation between bacteria, the immune response, and disease severity requires further studies.

The number of eosinophils was low in both groups, but there were still significantly more in dogs with rhinitis. We excluded dogs with predominantly eosinophilic inflammation since chronic eosinophilic rhinitis has been thought to be a separate disease entity related to type I hypersensitivity.^7,17,32^ Eosinophils have been described to be present in some of the dogs diagnosed with lymphoplasmacytic rhinitis and in human nonallergic rhinitis.^16,20,37^ The presence of small numbers of eosinophils in our population of dogs with rhinitis may be part of the inflammatory response or could indicate a possible allergic component to the disease. Interestingly, increased *interleukin-5* transcription without upregulation of other Th2 cytokines has been described in CIR dogs, which may play a role in antigen-induced tissue eosinophilia.^23^ The partial Th2 immune response detected in the nasal mucosa of dogs with CIR is similar to that in human nonallergic chronic rhinosinusitis, where eosinophils and lymphocytes are the predominant inflammatory cells.^23,27^ The number of eosinophils correlated with the increase in neutrophils and mucosal intraepithelial inflammation in our study and may be a part of the overall inflammatory response rather than specifically indicating allergic disease.

The number of mucosal intraepithelial leukocytes was significantly increased in dogs with CIR, which has also been reported in human chronic rhinosinusitis.^4^ In human nasal mucosa, intraepithelial and lamina propria lymphocytes have been suspected to comprise the regional immune system.^22^ The increase in intraepithelial leukocytes in diseased dogs could be triggered by microbes or other irritants in the nasal cavity. Intraepithelial lymphocytes were further evaluated with immunohistochemistry. T lymphocytes were detected both in the epithelium and the lamina propria, and B lymphocytes were only in the lamina propria, as described in the human nasal mucosa.^22^ Cytotoxic T-cells are found to predominate in the epithelium, being part of the innate immune reaction.^22^ Intraepithelial leukocytes represent a uniform but variable feature in diseased dogs that is only minimally detected in controls and represent typical changes in the inflammatory reaction. However, the inflammatory triggers in CIR dogs are unknown, and uncovering them is a goal for future research.

Intravascular neutrophils and vasculitis were significantly increased in dogs with CIR and have been described before in this disease.^37^ The presence of fibrosis and intravascular neutrophils are common but nonspecific findings in inflammatory reactions.^1^ The vasculitis detected in dogs with CIR is likely secondary to the nasal mucosal inflammatory process, as the clinical picture is not indicative of the primary immune-mediated vasculitis syndromes described in dogs, and secondary vasculitis is commonly described in a variety of inflammatory conditions including CIR.^31^ Lymphoid hyperplasia was equally detected in both groups and is likely to be a normal physiological finding.

In addition to inflammatory changes, we found mucosal epithelial alterations occurring in CIR. The epithelium was hyperplastic in dogs with CIR. Epithelial cells can undergo hyperplasia or metaplasia in response to chronic inflammation.^19^ Epithelial hyperplasia has been described before in dogs with CIR and in humans with chronic rhinosinusitis.^5,27,37^ We mainly detected epithelial hyperplasia in our study, but early metaplastic changes were also suspected in some of the biopsies, possibly explaining the loss of cilia and lack of goblet cells in these dogs, as squamous metaplasia of the respiratory epithelium can entail a loss of ciliated cells and goblet cells.^19^ Squamous metaplasia is described in human chronic rhinosinusitis.^4,21^ Chronic inflammatory rhinitis may have a duration of clinical signs extending from months to years,^37^ as seen in the presented cases. Interestingly, the only factor positively correlated with the duration of clinical signs was the presence of edema in the lamina propria. This was partially unexpected, as epithelial thickness or fibrosis could be connected to a longer duration of mucosal inflammation. This may be again partially explained by the population of CIR dogs with a relatively mild disease phenotype in this study.

The nasal epithelium provides a mechanical barrier against physical insult and inhaled particles but also has an additionally important role in innate immune functions.^28^ It has been shown in human nasal diseases that epithelial cells are actively involved in the inflammatory process by antigen presentation; pattern recognition functions; and by secreting cytokines, lipid mediators, and peptide products.^25,28,40^ Taking this into account, the inflammation in CIR may be originally initiated by epithelial cells in response to a stimulus, and the subsequent changes are due to epithelial cells producing pro-inflammatory cytokines and attracting and activating leukocytes. Epithelial cells can also contribute to chronic inflammatory reactions, eg, by the release of matrix metalloproteinase-9.^1^ Conversely, leukocytes release growth factors and cytokines^19,27^ that may result in the epithelial changes documented in CIR. The combination of these functions might lead to a vicious cycle that further promotes the loss of function of the nasal epithelium, resulting in clinical signs.

Fewer goblet cells were observed in dogs with CIR. Whether this is an actual decrease or whether increased mucus production has led to emptying of the goblet cells, making them less visible in the histological analysis, is yet to be determined. Our findings were contradictory to findings in human chronic rhinosinusitis, where goblet cell hyperplasia is often present.^4,21^ In our study, the number of goblet cells was negatively correlated with epithelial thickness, which could indicate the development of epithelial cell type changes in affected dogs. Some studies have not found a difference in the number of goblet cells between rhinitis and normal controls but have shown an increased functional activity in nasal goblet cells, explaining the increased secretion in rhinitis.^2,3^ In addition to goblet cells, nasal mucosal glands also contribute to mucus production.^7^ Further studies are needed to evaluate the presence and activity of goblet cells in dogs with CIR. In cases of actual decrease in goblet cells, the role of mucosal glands in increased mucus production in dogs with CIR should be further evaluated.

There was a loss of visible cilia in some of the dogs with rhinitis. The nasal biopsy samples of both groups were taken from approximately the same area in the nasal cavity, so the type of epithelium and presence of cilia are unlikely to be caused by sampling location. Cilia play a critical role in airway defense, as mucociliary clearance is an important means for eliminating microbes and other particles.^11^ Loss of cilia could contribute to the mucus accumulation seen clinically in these dogs and predispose to secondary infections. Loss of cilia has been described in human chronic rhinosinusitis due to squamous metaplasia of the respiratory epithelium.^4,21^ Further research is warranted to determine at which stage of the disease the loss of cilia occurs and if early detection and treatment of inflammation could prevent epithelial metaplasia and loss of cilia.

Although turbinate destruction is a common finding clinically, it was not detected in the histological evaluations. As almost all of the biopsies were obtained at the time of CT examination, the lack of cartilaginous/osseous changes is not explained by different time points in disease development. However, this evaluation was limited by superficial samples with only fragments of osseous/cartilaginous tissue present, and the results might have been different if more invasive surgical biopsies were obtained containing not only the mucosa but also largely the underlying cartilage and bone. In addition, as many biopsies were obtained in a blind fashion, the sampling may not have been targeted to the area of turbinate destruction. However, it is possible that the initial process leading to turbinate destruction is not present anymore at the time of the biopsy. Further research, including postmortem examination of CIR dogs with evaluation of the whole nasal cavity, could reveal if the inflammation reaches the cartilaginous and osseous tissue, which then leads to turbinate destruction.

This study had certain limitations. As an inherent limitation, the nasal biopsies obtained from clinical patients represented only a fraction of the entire nasal mucosa. In addition, there were inconsistencies in the size and quality of the biopsy samples. The small size of some of the biopsies and artifacts caused by stretching and crushing could have affected some of the analyses. This was most evident in the evaluation of edema in the lamina propria. For some of the specimens, it was only possible to evaluate 2 high-power fields instead of 3, which prompted mathematical extrapolation of cell count results in these cases. However, despite biopsy variations, the authors considered that they were able to assess all biopsies adequately. In addition, the retrospective identification of CIR dogs is a limitation. However, comprehensive evaluation was performed for all the dogs with the same protocol and at the same institution, with all the clinical data being available, both anterograde and retrograde rhinoscopy done, and the CT interpretation performed by a single radiologist, enabling us to exclude any focal lesions that might have been missed by evaluating only the biopsies. It is noteworthy that the dogs diagnosed with CIR had either mild or moderate clinical disease with mostly none to mild turbinate destruction. This may affect the histopathological changes detected in this study, and more severe changes may be detected in biopsies in a more severely affected population.

In conclusion, a small population of leukocytes was observed also in the nasal lamina propria of control dogs without nasal signs, likely representing a physiological immune cell population. Leukocyte numbers are increased in CIR, and the type of inflammation is not purely lymphoplasmacytic, as both neutrophils and eosinophils were also detected in CIR dogs. Significant epithelial alterations occur in CIR, including increased numbers of mucosal intraepithelial leukocytes and epithelial hyperplasia, which likely contribute to the clinical disease. A decrease in goblet cells and cilia was commonly detected in affected dogs and may represent a form of respiratory epithelial metaplasia.

## Supplemental Material

sj-pdf-1-vet-10.1177_03009858251349138 – Supplemental material for Nasal histological findings in asymptomatic control dogs and in dogs with chronic inflammatory rhinitis Supplemental material, sj-pdf-1-vet-10.1177_03009858251349138 for Nasal histological findings in asymptomatic control dogs and in dogs with chronic inflammatory rhinitis by Henriikka Neittaanmäki, Hanna-Maaria Javela, Essi Kuningas, Katja Koskinen, Anni Tilamaa, Minna Rajamäki, Sanna Viitanen and Niina Airas in Veterinary Pathology

sj-xlsx-2-vet-10.1177_03009858251349138 – Supplemental material for Nasal histological findings in asymptomatic control dogs and in dogs with chronic inflammatory rhinitis Supplemental material, sj-xlsx-2-vet-10.1177_03009858251349138 for Nasal histological findings in asymptomatic control dogs and in dogs with chronic inflammatory rhinitis by Henriikka Neittaanmäki, Hanna-Maaria Javela, Essi Kuningas, Katja Koskinen, Anni Tilamaa, Minna Rajamäki, Sanna Viitanen and Niina Airas in Veterinary Pathology
