# Supplementary material for: Nasal histological findings in asymptomatic control dogs and in dogs with chronic inflammatory rhinitis
Source: Vet Pathol. 2025 Jun 24;63(1):86–96. doi: 10.1177/03009858251349138 (PMC12743132; doi:10.1177/03009858251349138)
Supplement: sj-pdf-1-vet-10.1177_03009858251349138 – Supplemental material for Nasal histological findings in asymptomatic control dogs and in dogs with chronic inflammatory rhinitis [file sj-pdf-1-vet-10.1177_03009858251349138.pdf]

Supplemental Materials

Nasal histological findings in asymptomatic control dogs and in dogs with chronic inflammatory rhinitis

Henriikka Neittaanmäki, Hanna-Maaria Javela, Essi Kuningas, Katja Koskinen, Anni Tilamaa, Minna Rajamäki, Sanna Viitanen, Niina Airas

Supplemental Table S1. Detailed histopathological grading of the nasal biopsy specimens.

Supplemental Table S2. The histological grading of dogs with chronic inflammatory rhinitis and in control dogs without nasal signs. *See separate Excel file.*

Supplemental Table S3. Signalment and clinical signs of dogs with chronic inflammatory rhinitis (n=20).

Supplemental Table S4. Signalment, cause of euthanasia, and main postmortem diagnosis in control dogs without nasal signs (n=20).

Supplemental Table S1. Detailed histopathological grading of the nasal biopsy specimens.

Edema in the lamina propria

Nasal histological evaluation of edema in the lamina propria as **(a)** absent or **(b)** present in dogs with and without chronic inflammatory rhinitis. Hematoxylin and eosin. Grading based on percentage of the specimen area having edema as absent (0), mild (< 25 %), moderate (25-50 %), or severe (> 50 %).

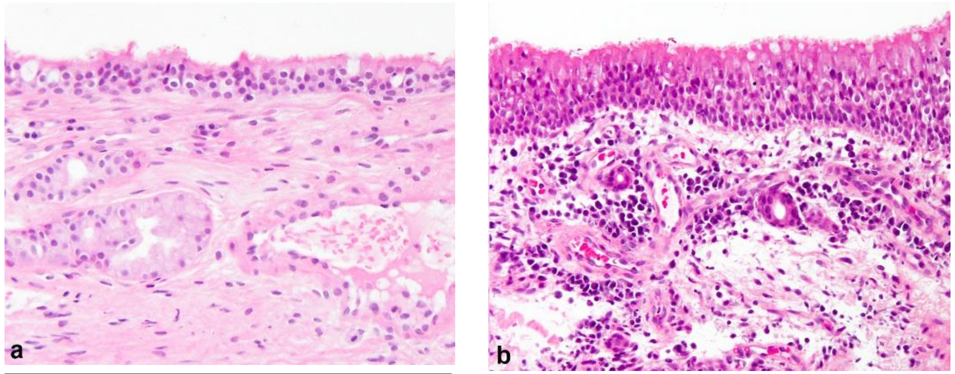

Area with edema absent

Area with edema present

Lymphoid hyperplasia

Nasal histological specimen from dog, lymphoid hyperplasia. Hematoxylin and eosin. Grading based on percentage of the specimen area having lymphoid hyperplasia as absent (0), mild (< 25 %), moderate (25-50 %), or severe (> 50 %).

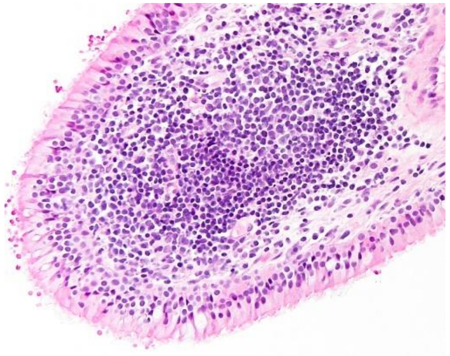

Area with lymphoid hyperplasia present

Fibrosis

The presence of fibrosis in nasal histological specimens from dogs with and without chronic inflammatory rhinitis, graded as **(a)** absent, **(b)** mild, **(c)** moderate and **(d)** severe based on the width of the fibrotic stroma and the presence of mucosal gland atrophy. Masson trichrome.

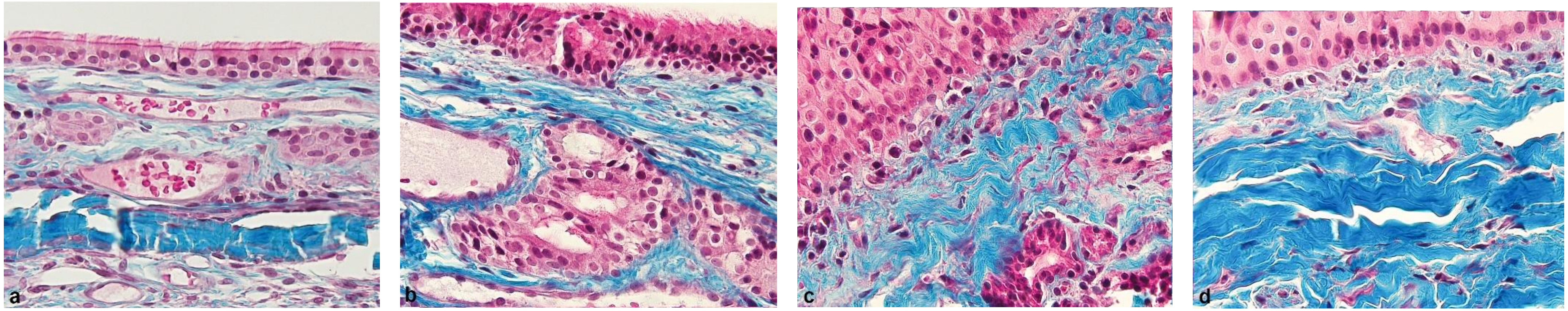

Absent: up to 2 fibroblasts width, normal mucosal glands

Mild: up to 5 fibroblasts width, normal mucosal glands

Moderate: up to 10 fibroblasts width, mild loss of mucosal glands

Severe: > 10 fibroblasts width, severe replacement of mucosal glands by fibrotic matrix

Epithelial damage

Epithelial damage in nasal histological specimens from dogs with and without chronic inflammatory rhinitis, graded as **(a)** absent, **(b)** erosion and **(c)** ulceration based on the severity of the epithelial damage. Hematoxylin and eosin.

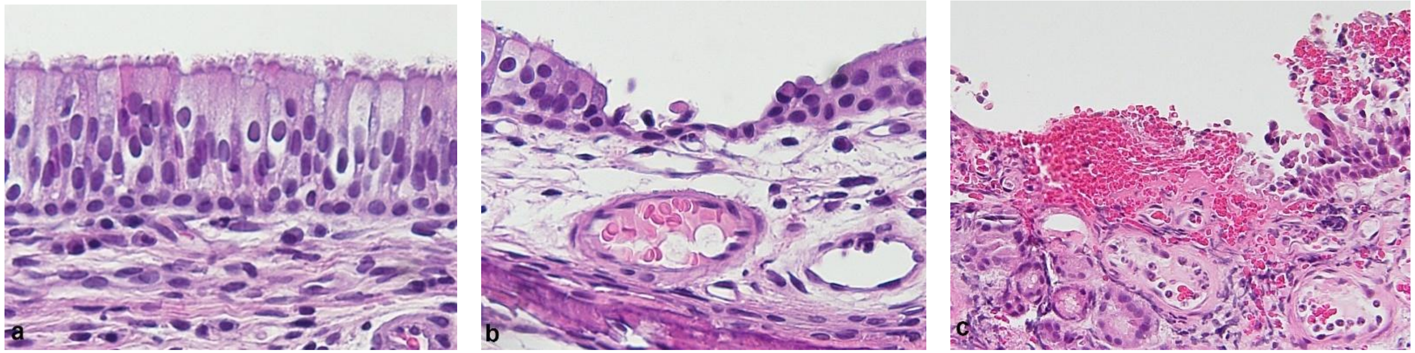

Absent: Normal epithelium

Erosion: Mild epithelial damage

Ulceration: damage extending through the basal cell layer and causing local inflammation

Goblet cells

The presence of visible goblet cells in nasal histological specimens from dogs with and without chronic inflammatory rhinitis, graded as **(a)** 0 (absent), **(b)** 1 (<5 per high-power field (HPF)), **(c)** 2 (5-10 per HPF), and **(d)** 3 (>10 per HPF) based on the frequency of visible goblet cells per HPF (0.237 mm^2) across the specimen. Periodic acid-Schiff.

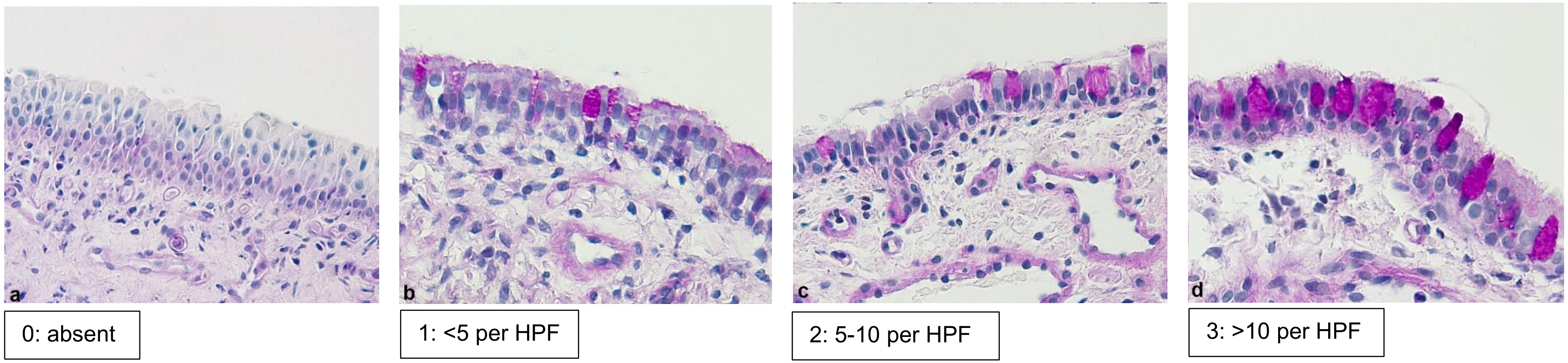

Supplemental Table S3. Signalment and clinical signs of dogs with chronic inflammatory rhinitis (n=20).

No.	Age (years)	Sex	Breed	Weight (kg)	Duration of clinical signs (months)	Nasal discharge (mucopurulent)	Nasal discharge (serous)	Sneezing	Reverse sneezing
1	9.5	FS	Parson Russell Terrier	9.2	8	x		x	x
2	10.6	FS	Mixed Breed	43	5	x		x	x
3	8	F	Parson Russell Terrier	6.4	6		x	x	
4	7.4	F	Miniature Pinscher	6.7	4	x		x	x
5	8	MN	American Akita	39.8	4	x	x	x	x
6	5.5	M	Dachshund	11.5	4	x	x	x	
7	7	FS	Dachshund	11.1	6	x		x	
8	2.5	M	Mixed Breed	32	12	x		x	
9	6	M	Briard	47.6	12	x		x	
10	8.2	F	Whippet	11.5	36	x		x	x
11	6	M	Parson Russell Terrier	7.1	8	x		x	x
12	3	M	Bordercollie	21.7	8	x		x	x
13	7.3	M	Dachshund	6	8	x	x	x	x
14	12	M	Dachshund	13.2	2	x			
15	7	M	Whippet	14	6	x		x	x
16	3.6	M	Mittelspitz	8.9	7	x		x	x
17	10.5	M	Dachshund	6.2	24	x		x	
18	11	FS	Parson Russell Terrier	9	12	x		x	
19	7	FS	Dachshund	7.4	8	x			x
20	10	M	Dachshund	8.4	36	x	x	x	x

M = male, MN = male neutered, F = female, FN = female spayed

Supplemental Table S4. Signalment, cause for euthanasia and main post-mortem diagnosis of control dogs without nasal signs (n=20).

No.	Age (years)	Sex	Breed	Weight (kg)	Cause for euthanasia	Post-mortem diagnosis (main)
1	9.2	M	Dachshund	12.2	Urinary signs, aggressiveness	Renal infarcts and cyst, prostatic hyperplasia
2	13.4	M	Mixed Breed	12.5	Acute deterioration, pain	Splenic hemangiosarcoma
3	4.8	M	Border collie	19.2	Aggressiveness	Spermatic granuloma (epididymis)
4	4.8	M	Karelian Bear Dog	27.5	Aggressiveness	Chronic lymphoplasmacytic thyroiditis
5	12.7	M	Grand Basset Griffon Vendéen	28.2	Acute deterioration, ascites	Prostatic adenocarcinoma, metastasis
6	13.8	MN	Border collie	15.3	Not known	Chronic follicular cystitis
7	4.5	M	Belgian Shepherd	32.0	Not known	No significant findings
8	11.5	FS	Borzoi	36.0	Chronic gastrointestinal signs	Hepatic histiocytic sarcoma
9	12.1	M	Giant Schnauzer	31.8	Hind limb paresis, pain	Pulmonary histiocytic sarcoma
10	11.7	MN	Mixed Breed	11.9	Suspected thyroid neoplasia	Thyroid adenocarcinoma
11	14.3	F	Keeshond	16.2	Vaginal and cervical mass	Cervical leiomyoma
12	9.0	F	Rottweiler	54.0	Osteoarthrosis	Osteoarthrosis
13	10.5	F	Icelandic Sheepdog	12.8	Liver and skin problems	Epidermal atrophy, interface dermatitis
14	11.8	MN	Golden Retriever	30.8	Not known	Osteoarthrosis
15	6.6	M	Cavalier King Charles Spaniel	8.0	Not known	Endocardosis, hydrocephalus
16	11.2	FS	Mixed Breed	26.4	Chronic vestibular signs	Cerebellar tumor (Gemistocytic astrocytoma)
17	16.8	M	Mixed Breed	32.8	Not known	Pulmonary local adenocarcinoma
18	7.2	FS	Mixed Breed	23.2	Life change of the owner	No significant findings
19	11.0	FS	Mixed Breed	10.0	Old age, health issues	No significant findings
20	12.5	FS	Dachshund	5.0	Old age, health issues	Thyroid atrophy

M = male, MN = male neutered, F = female, FN = female spayed
